# Minimally invasive surfactant administration in extremely preterm infants <28 weeks gestation with spontaneous breathing: a retrospective quality improvement study on reducing invasive ventilation burden

**DOI:** 10.3389/fped.2026.1780241

**Published:** 2026-03-16

**Authors:** Wenxin Dong, Hui Zhang, Xinyue Li, Hua Zhang, Zailing Li, Tongyan Han

**Affiliations:** 1 Department of Pediatrics, Peking University Third Hospital, Beijing, China; 2 Research Center of Clinical Epidemiology, Peking University Third Hospital, Beijing, China

**Keywords:** extremely preterm infants, invasive mechanical ventilation, minimally invasive surfactant administration, patent ductus arteriosus, quality improvement, respiratory distress syndrome

## Abstract

**Background:**

Invasive surfactant delivery via endotracheal intubation increases exposure to invasive mechanical ventilation (IMV) in extremely preterm infants (EPIs, <28 weeks). This quality improvement (QI) initiative aimed to evaluate whether minimally invasive surfactant administration (MISA) was associated with a reduced IMV burden in EPIs.

**Methods:**

A single-center retrospective QI study (2013–2024) included 115 infants (24–27⁺⁶ weeks) with spontaneous breathing diagnosed with respiratory distress syndrome (RDS). Controls (2013–2019, *n* = 55) received surfactant via intubation; the MISA group (2020–2024, *n* = 60) received surfactant via thin catheter during nasal continuous positive airway pressure/nasal intermittent positive pressure ventilation (NCPAP/NIPPV). Apart from the structured implementation of MISA, background respiratory and supportive care practices evolved gradually over time without other major structural changes. Multivariable regression and interrupted time-series analyses were performed to account for potential confounding and secular trends.

**Results:**

Baseline infant characteristics were comparable. The median duration of IMV within the first 72 h after birth was 0 h (IQR: 0–0) in the MISA group compared to 71.0 h (IQR: 19.0–72.0) in the control group (*P* < 0.001), and the difference remained significant after adjustment. Additionally, total IMV duration was reduced [0 h (IQR: 0–14) vs. 111 h (IQR: 39–264); *P* < 0.001], while non-invasive ventilation (NIV) duration was longer [51.5d (IQR: 41–57) vs. 37d (IQR 30–50); *P* < 0.001]. Lower incidences of hemodynamically significant patent ductus arteriosus (hsPDA) (35.0% vs. 69.1%; *P* < 0.001) and nosocomial pneumonia (18.9% vs. 67.9%; *P* < 0.001) were observed during the MISA implementation period, with consistent findings after multivariable adjustment. No significant differences were observed in severe intraventricular hemorrhage (IVH grade 3–4) (8.33% vs. 20.4%; *P* = 0.123), mortality (6.7% vs. 16.4%; *P* = 0.101), or other secondary outcomes (all *P* > 0.05). Interrupted time-series analysis demonstrated an immediate reduction in early IMV duration following MISA implementation, whereas changes in secondary outcomes did not reach statistical significance.

**Conclusion:**

MISA was associated with reduced early and overall IMV exposure in EPIs <28 weeks, and with lower observed incidences of hsPDA and nosocomial pneumonia. These associations warrant confirmation in prospective multicenter studies.

## Introduction

Respiratory distress syndrome (RDS) remains the predominant cause of morbidity and mortality in extremely preterm infants (EPIs) born at <28 weeks' gestation (1, 2). The pathophysiology centers on pulmonary surfactant deficiency compounded by structural lung immaturity. Conventional management requires endotracheal intubation for surfactant delivery coupled with invasive mechanical ventilation (IMV) (3). Although this approach improves respiratory failure, it inherently carries significant iatrogenic risks—notably bronchopulmonary dysplasia (BPD), intraventricular hemorrhage (IVH), and ventilator-induced lung injury (4). These complications disproportionately affect EPIs due to heightened vulnerability of their developing organs to invasive interventions (5).

Minimally invasive surfactant administration (MISA) has emerged as a paradigm-shifting alternative (6, 7). By administering surfactant via a thin catheter during non-invasive ventilation (NIV), MISA preserves spontaneous breathing, circumvents laryngeal trauma, and reduces IMV exposure (2, 8). Current evidence, however, predominantly supports its efficacy in preterm infants ≥28 weeks' gestation (3, 9, 10). Multicenter trials demonstrate reduced IMV duration in this cohort, yet subgroup analyses for infants <28 weeks remain underpowered due to limited sample sizes. Consequently, robust evidence regarding MISA's safety and effectiveness in the most vulnerable EPIs (<28 weeks)—a population with distinct physiological challenges including frequent respiratory failure and diminished tolerance to procedural stress—remains limited.

This relative paucity of specific data for the <28 weeks subgroup necessitates careful evaluation of MISA implementation in real-world settings, particularly within individual institutions. While quality improvement (QI) initiatives have refined respiratory support protocols in neonatal intensive care units (NICUs) (11, 12), the practical impact of MISA on reducing invasive ventilation burden specifically in EPIs <28 weeks warrants further examination based on institutional experience.

In our NICU, MISA has been progressively incorporated into the clinical pathway for spontaneously breathing preterm infants with RDS since 2020, building upon emerging evidence suggesting potential benefits in younger infants (3). This retrospective quality improvement study aimed to evaluate our institutional experience with MISA in EPIs. We compared outcomes between a historical cohort receiving surfactant via traditional endotracheal intubation and a subsequent cohort managed with the MISA approach. Our primary objective was to assess whether implementing MISA within our standardized QI framework was associated with changes in the duration of invasive mechanical ventilation within the critical first 72 h of life. Secondary objectives included exploring potential differences on other respiratory support durations and relevant complications. By analyzing this experience, we aimed to provide real-world data from a single-center QI initiative to inform surfactant administration strategies in this high-risk population and to offer contextual insights for similar clinical settings.

## Materials and methods

### Study design

This was a single-center, retrospective QI study conducted in NICU of Peking University Third Hospital from January 1, 2013, to December 31, 2024. The study compared two surfactant administration strategies:

**Control group (pre-QI period: 2013–2019)**: Surfactant delivery via endotracheal intubation with IMV.

**Intervention group (during QI implementation period: 2020–2024)**: MISA, involving surfactant delivery via a thin catheter during nasal continuous positive airway pressure (NCPAP) or nasal intermittent positive pressure ventilation (NIPPV)-assisted spontaneous breathing.

The QI initiative commenced on January 1, 2020, following the formation of a dedicated team (comprising three neonatologists) responsible for standardizing MISA protocols and facilitating staff training.

### Study population

**Inclusion criteria**:

Infants of 24 to 27^+6^ weeks' gestation.
Infants with spontaneous breaths and signs of respiratory distress that received non-invasive respiratory support immediately after birth in a delivery room and during transfer to the NICU.
Clinical diagnosis of RDS within the first 6 h of life. The diagnosis of RDS was based on clinical manifestations (respiratory rate >60/min, nasal flaring, retractions, grunting, or skin cyanosis) and [FiO_2_] > 0.3 for [SpO_2_] > 85%.

**Exclusion criteria**:

Infants meeting any of the following criteria were excluded:

Infants intubated prior to pulmonary surfactant administration due to postnatal
resuscitation or other reasons.
Infants with obvious malformations affecting respiratory function.
Infants transferred to other hospitals for surgery with incomplete data.
Infants withdrawn from the NICU by parents due to financial difficulties or poor prognosis.
Lack of complete medical records.

### Intervention: MISA protocol

This technique is also described in the literature as less invasive surfactant administration (LISA) or minimally invasive surfactant therapy (MIST).Although terminology varies, these terms refer to the same thin-catheter surfactant delivery approach during spontaneous breathing. In this manuscript, we use “MISA” for our institutional practice while retaining the original terminology when citing external studies. Throughout the 12-year study period (2013–2024), the general clinical management of extremely preterm infants in our unit evolved gradually in accordance with contemporaneous international and national guidelines, without abrupt structural changes in core respiratory or supportive care strategies. Delivery-room stabilization, early non-invasive respiratory support (primarily CPAP-based), routine caffeine administration, infection-prevention practices, nutritional support, and PDA management followed standardized institutional protocols that were updated incrementally over time in line with evolving evidence (13, 14). During the transition between periods, no additional structured respiratory quality-improvement programs were introduced apart from MISA. Within this relatively stable clinical framework, the implementation of the MISA protocol represented the principal structured quality-improvement intervention specifically targeting surfactant administration strategy.

### Standardized MISA procedure

Under NCPAP or NIPPV, the pulmonary surfactant was administered via MISA method within 6 h after birth if infants were clinically diagnosed with RDS. While the infants received NCPAP or NIPPV, a direct laryngoscope and a specifically designed thin catheter with 1.67 mm in diameter (catheter R, Beijing Double-crane pharmaceutical Co. LTD) were used to finish the surfactant administration. A direct laryngoscope was introduced to provide a glottal view before the operator inserts the catheter R 0.5–1.0 cm below the inverted V-shaped vocal cords. After withdrawing the laryngoscope, the operator fixed the thin catheter at the infant's mouth corner with the forefinger and thumb, and the end of the catheter R was connected to a 5 mL syringe containing surfactant solution. The surfactant at a dose of 100 mg/kg calf pulmonary surfactant (Beijing Double-Crane Pharmaceuticals Co. Ltd.) or a dose of 200 mg/kg Curosurf ® (Poractant alfa, Chiesi Farmaceutici, Italy) was administered through the catheter R by a nurse within 120–300 s by mini-boluses. The catheter R was immediately removed after surfactant administration. Infants remained on NCPAP or NIPPV post-administration unless meeting predefined intubation criteria (FiO_2_ > 0.4, pH: <7.20, or recurrent apnea).

All infants breathing spontaneously and those planned to be extubated soon received caffeine.

### Multiteam engagement

Weekly audits of surfactant administration practices.
Real-time feedback to clinicians on adherence to MISA protocols.
Monthly training sessions for NICU staff on catheter insertion techniques and complication management.

### Documentation and feedback

Electronic health records (EHRs) were updated to include MISA-specific fields (e.g., catheter placement attempts, adverse events).
Quarterly reports on MISA utilization rates and outcomes were reviewed by the QI team.

### Data collection and variables

Data were extracted retrospectively from EHRs using a structured template. The clinical data collected encompassed maternal and neonatal demographic characteristics (including birth weight, gestational age, sex, singleton/multiple gestation, Apgar scores at 1 and 5 min, antenatal corticosteroid administration), and in-hospital outcomes.

### Outcome measures

#### Primary outcome measure

The primary outcome was duration of IMV within 72 h after birth.

#### Secondary outcome measures

The secondary outcomes were as follows: requirement for≥2 doses of surfactant, BPD, IVH, periventricular leukomalacia (PVL), hemodynamically significant patent ductus arteriosus (hsPDA), pulmonary hemorrhage, pneumothorax, nosocomial pneumonia, late-onset sepsis (LOS) and severe retinopathy of prematurity (ROP, stage >2), in-hospital mortality, length of hospital stay, total duration of NIV, total duration of IMV, and days on supplemental oxygen.

### Disease diagnosis

BPD will be diagnosed according to the National Institutes of Health criteria (15). Pneumothorax will be diagnosed by the presence of air in the pleural cavity in a chest x-ray. Pulmonary hemorrhage will be diagnosed based on the gushing of bloody fluid from the upper airway or endotracheal intubation and when the chest x-ray is consistent with the relevant clinical findings. The diagnosis for hsPDA will be based on clinical signs and echocardiogram (16). IVH and PVL will be diagnosed by cranial ultrasound examination, and IVH will be graded by the Papile classification system (17). Nosocomial pneumonia developing ≥48 h after hospital admission with clinical and radiographic evidence. LOS will be defined as clinical sepsis with evidence of pathogens in blood culture. The diagnosis and staging of ROP will be based on retinal examination by a consultant ophthalmologist.

### Statistical analysis

Statistical analyses were performed using SPSS version 26.0 (IBM Corp.). Continuous data are presented as mean ± standard deviation for normally distributed variables or median with interquartile range (IQR) for non-normally distributed variables. Group comparisons for continuous variables utilized the Student's *t*-test (parametric) or the Mann–Whitney *U* test (non-parametric), based on distribution normality. Categorical variables are reported as frequencies (percentages), with group differences assessed using the chi-square test or Fisher's exact test for small cell counts.

To evaluate the independent effect of MISA, multivariable logistic and linear regressions were performed to adjust for confounders (gestational age, birth weight, sex, and antenatal steroids). Furthermore, Interrupted time-series (ITS) analysis was conducted based on annual data points (2013–2024, *n* = 12) to adjust for secular trends. Effect sizes are reported as odds ratios (ORs) or mean differences with 95% confidence intervals (CIs). Figures were generated using GraphPad Prism version 10.0. Given the retrospective and exploratory nature of this study, no formal adjustment for multiple comparisons (e.g., Bonferroni correction) was applied to the secondary outcomes. Two-tailed *p*-values <0.05 were considered significant; *p*-values reported as 0.000 by software were corrected to *p* < 0.001.

### Ethical considerations

The study was approved by the Institutional Review Board of Peking University Third Hospital (Protocol No: M20250673). Patient data were de-identified, and informed consent was waived due to the retrospective nature of the study.

## Results

### Trial population

**Figure 1:**
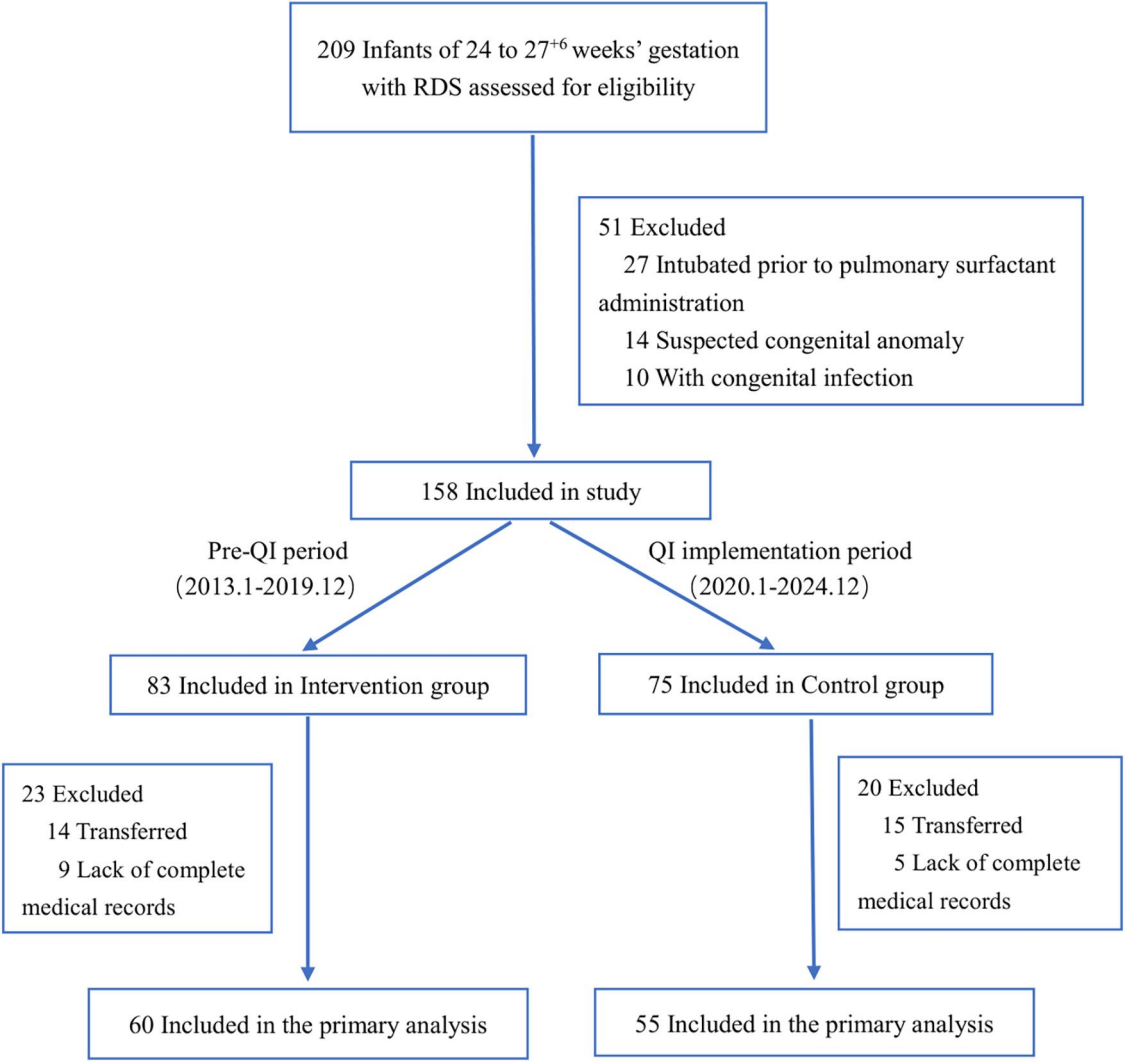
Patient recruitment flowchart. RDS, respiratory distress syndrome; QI, quality improvement.

A total of 209 infants born at 24–27^+6^ weeks' gestation with RDS were assessed for eligibility between January 2013 and December 2024 (Figure 1). After excluding 51 infants (27 intubated prior to surfactant administration, 14 with suspected congenital anomalies, and 10 with congenital infections), 158 infants were enrolled in the study. Following additional exclusions due to transfers and incomplete records, 115 infants were included in the final analysis: 60 in the intervention group (2020–2024 QI period) and 55 in the control group (2013–2019 pre-QI period).

### Baseline characteristics

Infant demographics and clinical characteristics were comparable between groups (Table 1). There were no significant differences in gestational age (26.8 ± 0.7 vs. 26.7 ± 0.7 weeks, *P* = 0.849), birth weight (953 ± 158 g vs. 979 ± 158 g, *P* = 0.387), sex distribution (45.0% vs. 54.5% male, *P* = 0.306), antenatal corticosteroid exposure (90.0% vs. 94.5%, *P* = 0.365), or Apgar scores indicative of asphyxia at 1 or 5 min (all *P* > 0.05).

**Table 1:** Infant demographic and clinical characteristics.

Variables	Intervention group (*n* = 60)	Control group (*n* = 55)	*t*/*χ*^2^	*P*
Gestational age at birth, mean (SD), wk	26.8 (0.7)	26.7 (0.7)	0.191	0.849
Birth weight, mean (SD), g	953.0 (158.0)	979.0 (158.0)	0.869	0.387
Male, No. (%)	27 (45.0)	30 (54.5)	1.050	0.306
Small-for-gestational-age birth weight, No. (%)	2 (3.33)	0 (0)		0.172*
Multiple birth, No. (%)	23 (38.3)	23 (41.8)	0.145	0.703
Exposure to any antenatal corticosteroids, No. (%)	54 (90.0)	52 (94.5)	0.822	0.365
Apgar score ≤3 at 1 min, No. (%)	0	0		1.000*
Apgar score ≤7 at 1 min, No. (%)	5 (8.3)	6 (10.9)	0.218	0.641
Apgar score ≤7 at 5 min, No. (%)	0	0		1.000*

Data are presented as mean ± standard deviation (SD), or number (%) where specified. Continuous variables were compared using student's *t*-test (parametric), and categorical variables were analyzed with *χ*^2^ test or Fisher's exact test.

* Fisher's exact test.

### Primary outcome

The median duration of IMV within the first 72 h after birth was 0 h (IQR: 0–0) in the intervention group compared to 71.0 h (IQR: 19.0–72.0) in the control group (*Z* = −7.351, *P* < 0.001) (Table 2). In multivariable linear regression analysis adjusting for gestational age, birth weight, sex, multiple gestation, antenatal steroid exposure, and 5 min Apgar score, the estimated difference in early IMV duration between groups remained statistically significant (adjusted *β* = 41.9 h, 95% CI: 30.9–52.8, *P* < 0.001) (Table 3).

**Table 2:** Comparison of primary and secondary outcomes between two groups.

Outcomes	Intervention group (*n* = 60)	Control group (*n* = 55)	*t*/*z*/*χ*^2^	Effect size (95% CI)	*P* value
	Primary outcome				
Duration of IMV within 72 h after birth, median (IQR), h	0 (0,0)	71.0 (19.0,72.0)	−7.351	MD −40.3 (−49.8 to −30.8)	<0.001
	Secondary outcomes				
Required ≥ 2doses of surfactant, No./total (%)	4/60 (6.67)	3/55 (5.45)		OR 1.24 (0.26–5.80)	0.549*
BPD grade in survivors at 36 wk, No./total (%)					
None	30/56 (53.6)	25/46 (54.3)	0.006	OR 0.97 (0.44–2.12)	0.938
Grade 1	18/56 (32.1)	12/46 (26.1)	0.188	OR 1.34 (0.57–3.19)	0.665
Grade 2	8/56 (14.3)	9/46 (19.6)	0.472	OR 0.69 (0.24–1.95)	0.492
Grade 3	0/56 (0)	0/46 (0)			1*
IVH, No./total (%)					
Grade 3–4	5/60 (8.33)	11/54 (20.4)	2.383	OR 0.36 (0.11–1.10)	0.123
Grade 1–2	55/60 (91.5)	43/54 (79.6)	0.542	OR 3.07 (1.00–9.38)	0.462
PVL, No./total (%)	4/60 (6.67)	4/54 (7.41)		OR 0.89 (0.21–3.76)	0.581*
hsPDA, No./total (%)	21/60 (35.0)	38/55 (69.1)	13.3	OR 0.24 (0.11–0.53)	<0.001
Pulmonary hemorrhage, No./total (%)	3/60 (5.00)	8/55 (14.5)		OR 0.31 (0.08–1.23)	0.077*
Pneumothorax, No./total (%)	1/60 (1.78)	0/55 (0)		OR 2.80 (0.11–70.14)	0.522*
Nosocomial pneumonia, No./total (%)	11/58 (18.9)	36/53 (67.9)	27.2	OR 0.11 (0.05–0.26)	<0.001
LOS, No./total (%)	14/59 (23.7)	14/53 (26.4)	0.107	OR 0.87 (0.37–2.04)	0.828
ROP (stage >2), No./total (%)	4/56 (7.1)	1/46 (2.17)		OR 3.46 (0.37–32.06)	0.369*
In-hospital mortality, No./total (%)	4/60 (6.67)	9/55 (16.4)	2.69	OR 0.37 (0.11–1.29)	0.101
	Alive at hospital discharge				
Hospitalization days, mean (SD), d	72.6 (15.5)	68.5 (19.7)	1.204	MD 3.8 (−2.3 to 10.0)	0.232
Total duration of IMV, median (IQR), h	0 (0, 14.0)	111.0 (38.7, 264.0)	−6.381	MD −187.5 (−259.1 to −115.9)	<0.001
Total duration of NIV, median (IQR), d	51.5 (41.0, 57.0)	37.0 (30, 50.3)	−3.464	MD 10.1 (5.1–15.1)	<0.001
Duration of supplemental oxygen, mean (SD), d	59.7 (15.1)	60.1 (20.4)	−0.142	MD 1.1 (−5.4 to 7.6)	0.887

BPD, bronchopulmonary dysplasia; hsPDA, hemodynamically significant patent ductus arteriosus; IMV, invasive mechanical ventilation; NIV, non-invasive ventilation; IVH, intraventricular hemorrhage; LOS, late-onset sepsis; PVL, periventricular leukomalacia; ROP, retinopathy of prematurity. Data are presented as median [interquartile range (IQR)], number (%), or mean ± standard deviation (SD) where specified. Group comparisons used Mann–Whitney *U* test (*Z*-values) or Student's *t*-test for continuous variables and *χ*^2^ test for categorical variables. Effect sizes are reported as Odds Ratios (OR) for categorical outcomes and Mean Differences (MD) for continuous outcomes, with 95% confidence intervals (CIs).

* Fisher's exact test.

**Table 3:** Multivariable regression analysis of primary and secondary outcomes.

Outcome	Adjusted *β* (95% CI)	Adjusted OR (95% CI)	*P* value
Continuous outcomes			
Duration of IMV within 72 h (h)	−41.9 (−52.8 to −30.9)	—	<0.001
Total duration of IMV (h)	−148.6 (−220.7 to −76.5)	—	<0.001
Total duration of NIV (days)	12.0 (6.0–18.1)	—	<0.001
Duration of supplemental oxygen (days)	−2.8 (−11.0 to 5.4)	—	0.50
Binary outcomes			
Nosocomial pneumonia	—	0.084 (0.029–0.244)	<0.001
hsPDA	—	0.290 (0.106–0.794)	0.016
Late-onset sepsis	—	1.01 (0.356–2.857)	0.99
In-hospital mortality	—	0.306 (0.072–1.299)	0.11
Severe IVH (Grade III–IV)	—	0.429 (0.116–1.587)	0.21
Pulmonary hemorrhage	—	0.275 (0.056–1.351)	0.112
PVL	—	1.85 (0.25–13.51)	0.548
Required ≥ 2 doses of surfactant	—	0.91 (0.162–5.00)	0.912
BPD (Any grade)	—	1.69 (0.699–4.17)	0.242

IMV, invasive mechanical ventilation; NIV, non-invasive ventilation; hsPDA, hemodynamically significant patent ductus arteriosus; IVH, intraventricular hemorrhage; PVL, periventricular leukomalacia; BPD, bronchopulmonary dysplasia. Data are presented as adjusted *β* coefficients or adjusted odds ratios (aORs) with 95% confidence intervals (CIs). Continuous outcomes were analyzed using linear regression and binary outcomes using logistic regression. All models were adjusted for gestational age, birth weight, sex, antenatal steroid exposure, multiple gestation, and 5-min Apgar score. The Control group served as the reference category.

### Secondary outcomes

Total IMV duration was reduced [0 h (IQR: 0–14) vs. 111 h (IQR: 39–264); *P* < 0.001], while NIV duration was longer [51.5d (IQR: 41–57) vs. 37d (IQR: 30–50); *P* < 0.001] in the intervention group compared to the control group. After adjustment for baseline characteristics, these differences remained statistically significant (IMV: adjusted *β* = −148.6, 95% CI: −220.7 to −76.5, *P* < 0.001; NIV: adjusted *β* = 12.0, 95% CI: 6.0–18.1, *P* < 0.001) (Tables 2, 3).

The incidence of hsPDA was lower in the intervention group compared with the control group (35.0% vs. 69.1%; *P* < 0.001). In the adjusted logistic regression model, the between-group difference was statistically significant (adjusted OR = 0.290, 95% CI: 0.106–0.794, *P* = 0.016). Nosocomial pneumonia occurred less frequently in the intervention group (18.9% vs. 67.9%; *P* < 0.001). The adjusted analysis yielded a statistically significant between-group difference (adjusted OR = 0.084, 95% CI: 0.029–0.244, *P* < 0.001) (Tables 2, 3).

Additionally, no significant between-group differences were observed for severe IVH, mortality, requirement for ≥2 doses of surfactant, BPD severity, PVL, pulmonary hemorrhage, LOS, severe ROP, duration of supplemental oxygen, or length of hospital stay (all *P* > 0.05). After multivariable adjustment, these findings remained unchanged, and no statistically significant associations were identified for these outcomes (Tables 2, 3).

The analysis of BPD and hospitalization outcomes was restricted to infants who survived to discharge. Analyses for LOS and nosocomial pneumonia excluded three infants who died within 72 h, with one of these also excluded from IVH and PVL assessments due to lacking cranial ultrasound.

### Interrupted time-series analysis

**Figure 2:**
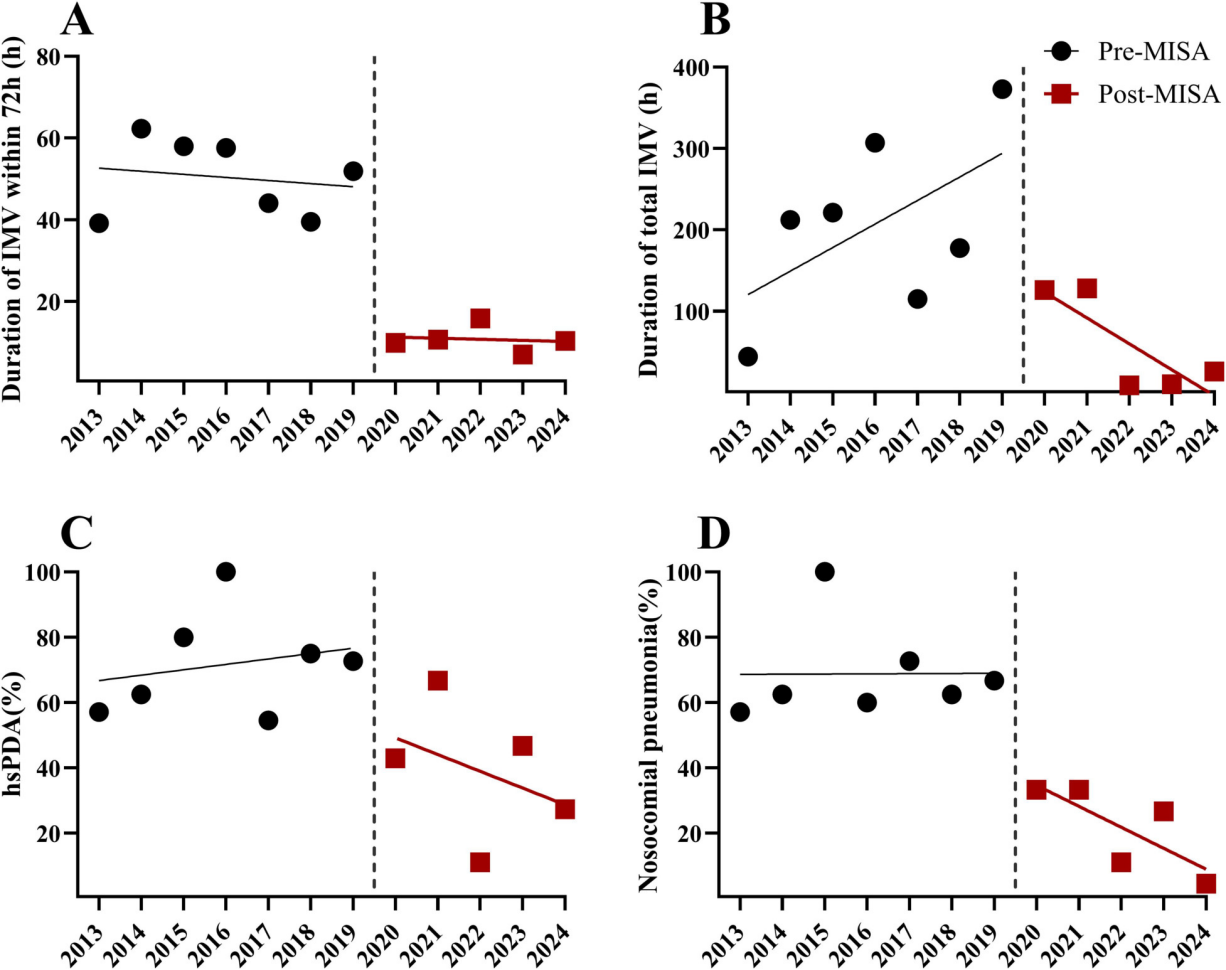
Interrupted time series analysis of key outcomes **(A)** IMV duration within 72 h. **(B)** Total IMV duration. **(C)** Incidence of hsPDA. **(D)** Incidence of nosocomial pneumonia. The vertical dashed line marks the MISA implementation. Circles and squares denote observed pre- and post-intervention data, respectively; solid lines represent regression trends. IMV, invasive mechanical ventilation; hsPDA, hemodynamically significant patent ductus arteriosus; MISA, minimally invasive surfactant administration.

The results of the ITS analysis are presented in Table 4 and Figure 2. While the ITS model included annual time points, the relatively small sample size per year may have contributed to fluctuations in the estimates. Following implementation of MISA, an immediate level change in early IMV duration was estimated (*β* = −36.05 h, 95% CI: −58.21 to −13.90, *P* = 0.006). No statistically significant baseline pre-intervention trend was detected. For total IMV duration, a change in slope was estimated (*β* = −199.59, 95% CI: −419.48 to 20.30, *P* = 0.070), although the result did not reach statistical significance. For hsPDA and nosocomial pneumonia, regression coefficients showed changes in the same direction as those observed in the cohort analysis; however, these did not reach statistical significance in the ITS model (all *P* > 0.05).

**Table 4:** Interrupted time series analysis of Key outcomes.

Outcome	Baseline trend (β, 95% CI)	*P* value	Immediate level change (β, 95% CI)	*P* value	Change in slope (β, 95% CI)	*P* value
Continuous outcomes						
Duration of IMV within 72 h	−0.76 (−4.41 to 2.90)	0.65	−36.05 (−58.21 to −13.90)	0.006	0.49 (−6.63 to 7.61)	0.88
Total duration of IMV	28.96 (−7.29 to 65.21)	0.10	−199.59 (−419.48 to 20.30)	0.070	−60.80 (−131.46 to 9.86)	0.083
Binary outcomes (%)						
Nosocomial pneumonia	0.05 (−6.07 to 6.16)	0.99	−34.33 (−71.42 to 2.76)	0.065	−6.47 (−18.39 to 5.45)	0.25
hsPDA	1.65 (−6.63 to 9.93)	0.66	−29.17 (−79.40 to 21.07)	0.22	−6.77 (−22.91 to 9.37)	0.36

IMV, invasive mechanical ventilation; hsPDA, hemodynamically significant patent ductus arteriosus. Interrupted time series analysis was performed using segmented linear regression models. Baseline trend represents the pre-intervention slope; immediate level change represents the change in outcome immediately after implementation of the intervention; change in slope represents the difference between pre- and post-intervention trends. Data are presented as regression coefficients (β) with 95% confidence intervals (CIs).

## Discussion

This retrospective quality improvement study evaluated the integration of MISA into the management pathway for RDS in spontaneously breathing EPIs under 28 weeks of gestation within a standardized framework. The baseline characteristics, including gestational age, birth weight, antenatal corticosteroid exposure, and Apgar scores indicative of perinatal asphyxia at 1 and 5 min, were comparable between the two groups. Compared to the historical control group receiving surfactant via endotracheal intubation, MISA implementation was associated with shorter IMV duration within the first 72 h and lower incidences of hsPDA and nosocomial pneumonia, with findings remaining consistent after multivariable adjustment. In the ITS analysis, only early IMV showed a significant immediate reduction, whereas hsPDA and nosocomial pneumonia demonstrated similar directional trends without statistical significance. Nevertheless, these observations should be considered exploratory and interpreted with caution given the relatively small number of patients per year and the inherent limitations of the historical-control design with potential residual confounding.

The primary finding of this study—that MISA was associated with a reduced burden of mechanical ventilation within 72 h and overall—aligns with the conclusions of several previous studies involving similar gestational age groups, including those by Boskabadi et al. (26–34 weeks) (18), Anand et al. (26–34 weeks) (19), Dargaville et al. (25–28 weeks) (20), Kaleem et al. (<34 weeks) (21), and Wang et al. (<32 weeks) (22). Furthermore, a meta-analysis by Abdel-Latif et al., which pooled evidence from 16 randomized trials, reported that the MISA method reduced the risk of death or BPD, decreased intubation within 72 h, and lowered the incidence of major complications and in-hospital mortality (23). This beneficial effect may stem from MISA's dual protective mechanisms: avoiding the stress of intubation and eliminating the injury associated with early positive pressure ventilation. Animal studies suggest that mechanical ventilation can impair the initial lung tissue association of exogenous surfactant, linked to reduced dynamic compliance and increased surfactant inactivation. In contrast, the surfactant distribution process during MISA is likely more physiological (18, 24–26).

Regarding secondary outcomes, the MISA group was associated with a lower incidence of hsPDA, aligning with findings from previous studies (3). This observed association may be related to the avoidance of invasive positive pressure ventilation and its associated hemodynamic fluctuations. First, MISA preserves spontaneous breathing and physiological intrathoracic pressure, which avoids the compromised systemic venous return and hemodynamic instability often linked to invasive ventilation—factors known to influence ductal patency. Second, by minimizing procedure-related hypoxia and mechanical lung injury, MISA may help maintain stable arterial oxygen tension and reduce the release of inflammatory mediators (e.g., prostaglandins), both of which are critical for facilitating ductal constriction (18, 27, 28). The incidence of nosocomial pneumonia was also lower in the MISA group. Boskabadi et al. similarly reported reduced infection-related complications in their MIST group (18). This observed association may be partially explained by avoiding endotracheal intubation and significantly shortening IMV duration, thereby directly reducing exposure to the risk of VAP. Interestingly, the incidence of LOS remained comparable between the two groups. While the continuous refinement of neonatal intensive care practices over the study period undoubtedly improved overall clinical safety, the observed reduction in pneumonia—in the context of stable LOS rates—suggests that the implementation of MISA may have provided an additional, targeted advantage in protecting the vulnerable respiratory tract of these extremely preterm infants.

In the present study, no significant differences were observed between groups for other outcomes, including severe IVH, mortality, BPD, PVL, pulmonary hemorrhage, LOS, severe ROP, or length of hospital stay. Specifically, our study may have been underpowered to detect differences in mortality and severe IVH due to the small sample size. Additionally, the absence of severe BPD cases may be attributed to our inclusion criteria and a potential survival bias, as the most critical infants might not have survived to the assessment age. Our findings regarding secondary complications are consistent with the heterogeneity observed in existing literature. For instance, while the OPTIMIST-A trial reported a reduced incidence of BPD with LISA (20), other studies and a systematic review by Kuitunen et al. found mixed results or no significant differences in outcomes such as IVH and pneumothorax (29–31). The heterogeneity in reported outcomes for these complications across the literature may be attributed to the inherently high-risk profile of EPIs. The elevated baseline rate of severe morbidities in infants under 28 weeks can dilute the measurable effect of a single intervention, thereby complicating the interpretation of MISA's independent contribution. Furthermore, differences in study design, sample size, and background care practices likely contribute to these discrepant findings.

This study involved a trade-off associated with the change in respiratory support mode. While MISA shortened IMV duration and avoided its direct risks (barotrauma, VAP), it was associated with a prolonged duration of NIV, which could potentially increase the risks of nasal injury and high oxygen requirements. A key strength of this study is its focus on the EPI subgroup under 28 weeks, for which evidence is scarce, providing high-quality real-world data. The implementation within a standardized QI framework—incorporating defined procedures, training, and auditing—enhances the reproducibility of this practice.

Nevertheless, this study has several limitations. The retrospective, historical control design cannot entirely separate the independent effect of MISA from the secular trends in NICU care over the 12-year period. We acknowledge that clinical protocols and respiratory management evolved progressively, which may have contributed to the improved outcomes. Although we employed multivariable regression and ITS analysis to mitigate these effects, residual confounding may still remain. Furthermore, the single-center nature limits generalizability, and the relatively small sample size may have underpowered our ability to detect differences in less frequent outcomes, such as mortality and severe IVH. To confirm these preliminary findings, prospective parallel-controlled trials or multi-center contemporaneous comparisons are warranted.

## Conclusion

This study indicates that the implementation of MISA, supported by a systematic quality improvement framework, is associated with a significant reduction in both early and total invasive mechanical ventilation burden in extremely preterm infants under 28 weeks of gestation. It is also associated with a decreased incidence of hemodynamically significant PDA and nosocomial pneumonia, without evidence of increased major short-term risks in this cohort. These findings support MISA as a feasible and potentially beneficial strategy for reducing exposure to invasive ventilation in this vulnerable population. However, the prolonged duration of non-invasive ventilation (NIV) observed during the MISA period represents a potential trade-off that warrants further evaluation. Given the inherent limitations of the retrospective, single-center, historical-control design, these findings should be interpreted as associations rather than definitive causal links, as residual confounding from secular trends in neonatal intensive care practices cannot be entirely excluded. Furthermore, the impact of MISA on long-term neurodevelopmental and pulmonary outcomes requires validation through large-scale, prospective, multicenter randomized controlled trials. Continual optimization of MISA procedures and integration of robust quality improvement initiatives remain essential.

## Data Availability

The data analyzed in this study is subject to the following licenses/restrictions: The datasets are available from the corresponding author on reasonable request. Requests to access these datasets should be directed to tongyanhan@bjmu.edu.cn.

## References

[1] Alsina-Casanova M Brito N Balcells-Esponera C Herranz-Barbero A Teresa-Palacio M Soler-García A Predictors of CPAP failure after less-invasive surfactant administration in preterm infants. Front Pediatr. (2024) 12:1444906. 10.3389/fped.2024.1444906 39258148 PMC11383777

[2] Heiring C Hedegaard SS Carlsen EM Kristensen R Breindahl N Schmidt C Less invasive surfactant administration versus intubate-surfactant-extubate: associated with reduced mechanical ventilation in extremely preterm infants. Acta Paediatr. (2025). 10.1111/apa.70041 PMC12258129 40008543

[3] Han T Liu H Zhang H Guo M Zhang X Duan Y Minimally invasive surfactant administration for the treatment of neonatal respiratory distress syndrome: a multicenter randomized study in China. Front Pediatr. (2020) 8:182. 10.3389/fped.2020.00182 PMC7221055 32457854

[4] Dini G Santini MG Celi F Verrotti A. Less invasive surfactant administration versus intubation-surfactant-extubation in preterm infants: a retrospective study. Med Glas (Zenica). (2024) 21(2):309–14. 39526720 10.17392/1726-21-02

[5] Edwards EM Ehret DEY Cohen H Zayack D Soll RF Horbar JD. Quality improvement interventions to prevent intraventricular hemorrhage: a systematic review. Pediatrics. (2024) 154(2):e2023064431. 10.1542/peds.2023-064431 38982935

[6] Breindahl N Henriksen TB Heiring C Bay ET Haaber J Salmonsen TG NON-pharmacological approach less invasive surfactant administration (NONA-LISA) trial: protocol for a randomised controlled trial. Pediatr Res. (2024) 96(4):1084–9. 10.1038/s41390-023-02998-0 38200325 PMC11502479

[7] Buyuktiryaki M Alarcon-Martinez T Simsek GK Canpolat FE Tayman C Oguz SS Five-year single center experience on surfactant treatment in preterm infants with respiratory distress syndrome: LISA vs INSURE. Early Hum Dev. (2019) 135:32–6. 10.1016/j.earlhumdev.2019.06.004 31229792

[8] Federici C Fornaro G Roehr CC. Cost-saving effect of early less invasive surfactant administration versus continuous positive airway pressure therapy alone for preterm infants with respiratory distress syndrome. Eur J Hosp Pharm. (2022) 29(6):346–52. 10.1136/ejhpharm-2020-002465 33658228 PMC9614139

[9] Elbaz Y Portnov I Lurie-Marcu B Shinwell ES. Minimally invasive surfactant therapy versus intubation for surfactant delivery in preterm infant with RDS: evaluation of safety and efficacy. J Matern Fetal Neonatal Med. (2022) 35(25):6802–6. 10.1080/14767058.2021.1924145 34024234

[10] Herting E Kribs A Härtel C von der Wense A Weller U Hoehn T Two-year outcome data suggest that less invasive surfactant administration (LISA) is safe. Results from the follow-up of the randomized controlled AMV (avoid mechanical ventilation) study. Eur J Pediatr. (2020) 179(8):1309–13. 10.1007/s00431-020-03572-0 32067100 PMC7351829

[11] Alshaikh BN Sproat TDR Wood C Spence J-M Knauff M Hamilton C A quality improvement initiative to reduce necrotizing enterocolitis in very preterm infants. Pediatrics. (2023) 152(6):e2023061273. 10.1542/peds.2023-061273 37920940

[12] Berneau P Nguyen Phuc Thu T Pladys P Beuchée A. Impact of surfactant administration through a thin catheter in the delivery room: a quality control chart analysis coupled with a propensity score matched cohort study in preterm infants. PLoS One. (2018) 13(12):e0208252. 10.1371/journal.pone.0208252 30540816 PMC6291238

[13] Sweet DG Carnielli VP Greisen G Hallman M Klebermass-Schrehof K Ozek E European Consensus guidelines on the management of respiratory distress syndrome: 2022 update. Neonatology. (2023) 120(1):3–23. 10.1159/000528914 36863329 PMC10064400

[14] Aziz K Lee HC Escobedo MB Hoover AV Kamath-Rayne BD Kapadia VS Part 5: neonatal resuscitation: 2020 American Heart Association guidelines for cardiopulmonary resuscitation and emergency cardiovascular care. Circulation. (2020) 142(16 Suppl 2):S524–50. 10.1161/CIR.0000000000000902 33081528

[15] Jobe AH Bancalari E. Bronchopulmonary dysplasia. Am J Respir Crit Care Med. (2001) 163(7):1723–9. 10.1164/ajrccm.163.7.2011060 11401896

[16] Hamrick SEG Sallmon H Rose AT Porras D Shelton EL Reese J Patent ductus arteriosus of the preterm infant. Pediatrics. (2020) 146(5):e20201209. 10.1542/peds.2020-1209 33093140 PMC7605084

[17] Papile LA Burstein J Burstein R Koffler H. Incidence and evolution of subependymal and intraventricular hemorrhage: a study of infants with birth weights less than 1,500 gm. J Pediatr. (1978) 92(4):529–34. 10.1016/S0022-3476(78)80282-0 305471

[18] Boskabadi H Behmadi M Maamouri G Loghmani T Rangrazi A. Comparing the effects of two surfactant administration methods: minimally invasive surfactant therapy (MIST) with intubation (INSURE) in infants with respiratory distress syndrome. Adv Respir Med. (2024) 92(5):384–94. 10.3390/arm92050036 39452058 PMC11505403

[19] Anand R Nangia S Kumar G Mohan MV Dudeja A. Less invasive surfactant administration via infant feeding tube versus InSurE method in preterm infants: a randomized control trial. Sci Rep. (2022) 12(1):21955. 10.1038/s41598-022-23557-3 36535971 PMC9763238

[20] Dargaville PA Kamlin COF Orsini F Wang X De Paoli AG Kanmaz Kutman HG Effect of minimally invasive surfactant therapy vs sham treatment on death or bronchopulmonary dysplasia in preterm infants with respiratory distress syndrome: the OPTIMIST-A randomized clinical trial. Jama. (2021) 326(24):2478–87. 10.1001/jama.2021.21892 34902013 PMC8715350

[21] Kaleem A Haroon F Fatima B Victor G Qadir M Waheed KAI. Efficacy and safety of surfactant administration by MIST and INSURE techniques in neonates with respiratory distress syndrome: a randomized controlled trial. Pak J Med Sci. (2023) 39(3):848–52. 10.12669/pjms.39.3.7283 37250559 PMC10214821

[22] Wang XA Chen LJ Chen SM Su P-H Chen J-Y. Minimally invasive surfactant therapy versus intubation for surfactant administration in very low birth weight infants with respiratory distress syndrome. Pediatr Neonatol. (2020) 61(2):210–5. 10.1016/j.pedneo.2019.11.002 31818537

[23] Abdel-Latif ME Davis PG Wheeler KI De Paoli AG Peter A. Surfactant therapy via thin catheter in preterm infants with or at risk of respiratory distress syndrome. Cochrane Database Syst Rev. (2021) 5(5):CD011672. 10.1002/14651858.CD011672.pub2 33970483 PMC8109227

[24] Bohlin K Bouhafs RK Jarstrand C Curstedt T Blennow M Robertson B. Spontaneous breathing or mechanical ventilation alters lung compliance and tissue association of exogenous surfactant in preterm newborn rabbits. Pediatr Res. (2005) 57(5 Pt 1):624–30. 10.1203/01.PDR.0000156502.84909.BC 15718361

[25] Niemarkt HJ Kuypers E Jellema R Ophelders D Hütten M Nikiforou M Effects of less-invasive surfactant administration on oxygenation, pulmonary surfactant distribution, and lung compliance in spontaneously breathing preterm lambs. Pediatr Res. (2014) 76(2):166–70. 10.1038/pr.2014.66 24796373

[26] Ricci F Bresesti I LaVerde PAM Salomone F Casiraghi C Mersanne A Surfactant lung delivery with LISA and InSurE in adult rabbits with respiratory distress. Pediatr Res. (2021) 90(3):576–83. 10.1038/s41390-020-01324-2 33452472 PMC7809896

[27] Willis KA Weems MF. Hemodynamically significant patent ductus arteriosus and the development of bronchopulmonary dysplasia. Congenit Heart Dis. (2019) 14(1):27–32. 10.1111/chd.12691 30343505

[28] van Vonderen JJ Roest AA Siew ML Blom NA van Lith JM Walther FJ Noninvasive measurements of hemodynamic transition directly after birth. Pediatr Res. (2014) 75(3):448–52. 10.1038/pr.2013.241 24346112

[29] Mehler K Broer A Roll C Göpel W Wieg C Jahn P Developmental outcome of extremely preterm infants is improved after less invasive surfactant application: developmental outcome after LISA. Acta Paediatr. (2021) 110(3):818–25. 10.1111/apa.15565 32892376

[30] Pérez-Iranzo A Jarque A Toledo JD Tosca R. Less invasive surfactant administration reduces incidence of severe intraventricular haemorrage in preterms with respiratory distress syndrome: a cohort study. J Perinatol. (2020) 40(8):1185–92. 10.1038/s41372-020-0702-5 32546828

[31] Kuitunen I Räsänen K. Less invasive surfactant administration compared to intubation, surfactant, rapid extubation method in preterm neonates: an Umbrella review. Neonatology. (2024) 121(4):485–93. 10.1159/000537903 38503270 PMC11318579

